# A case of autosomal dominant polycystic kidney disease with multiple bilateral spontaneous retroperitoneal hemorrhages

**DOI:** 10.1007/s13730-025-01079-x

**Published:** 2026-03-09

**Authors:** Masato Mizuta, Tatsuya Suwabe, Yuki Oba, Akinari Sekine, Masayuki Yamanouchi, Hiroki Mizuno, Eiko Hasegawa, Takehiko Wada, Makoto Fukuda, Motoaki Miyazono, Naoki Sawa, Yoshifumi Ubara

**Affiliations:** 1 Nephrology Center and Okinaka Memorial Institute for Medical Research, Toranomon Hospital Kajigaya, Kawasaki, Kanagawa Japan; 2 Department of Nephrology, Saga University Internal Medicine, Saga, Japan; 3 Department of Nephrology, Toranomon Hospital Kajigaya, 1-3-1, Takatsu, Kawasaki, Kanagawa 213-8587 Japan

**Keywords:** Bilateral spontaneous retroperitoneal bleeding, Autosomal polycystic kidney disease (ADPKD), Subcapsular renal hematoma, Perinephric hematoma, Transarterial embolization (TAE)

## Abstract

We experienced a 45-year-old male with ADPKD who developed spontaneous retroperitoneal hemorrhages with hypertension in the right subcapsular renal space without trauma. After his condition worsened with conservative management, he was treated by renal transarterial embolization (TAE). Over the next eight years, he experienced bilateral bleeding at three different sites and underwent repeated endovascular treatment with renal TAE to stop the bleeding. After the fourth occurrence, the patient underwent hemodialysis. Although renal cysts and urinary tract bleeding occur frequently in ADPKD, retroperitoneal hemorrhage is rare; however, TAE is both effective and a good way to preserve renal function.

## Introduction

Hematoma due to bleeding in the space between the renal fascia (Gerota's fascia) and the renal capsule is called subcapsular renal hematoma or perinephric hematoma. This type of hematoma compresses the renal parenchyma and activates the renin–angiotensin–aldosterone system, resulting in systemic hypertension. This condition, called page kidney, is most commonly caused by sports injuries and hemorrhage from renal biopsies [1–5]. In the past, it was treated by surgical nephrectomy.

There have been very few reports on page kidney in autosomal dominant polycystic kidney disease (ADPKD), which is known to be prone to renal hemorrhage. Here, we present a case of repeated subcapsular hematoma without any obvious cause, such as trauma, in which renal function was maintained by selective transarterial embolization (TAE) of renal capsular arteries with microcoils.

## Case report

A 45-year-old man was first admitted to our hospital because of worsening right-flank pain that had developed without trauma or other triggers.His mother had previously been diagnosed with the same disease.

The patient was diagnosed with ADPKD at the age of 40 when he visited a local clinic complaining of abdominal pain.At that time, the patient had a creatinine level of 1.0 mg/dL (eGFR 67.3 mL/min/1.73 m^2^), and hypertension (150/90 mmHg) was noted, which was managed with calcium channel blockers alone. The abdominal pain improved with observation alone. Subsequently, the patient occasionally experienced episodes of abdominal pain, but all symptoms improved with observation alone.

One week prior to admission to our hospital, the patient presented with right abdominal pain and visited a nearby clinic. This episode was the most intense attack of abdominal pain he had ever experienced. He was diagnosed with subcapsular hemorrhage of the right upper kidney. At that time, his creatinine level was 1.3 mg/dL and hemoglobin level was 15.9 g/dL. Simultaneously, he developed hypertension (190/110 mmHg) with hyperreninemia (renin activity 12 ng/mL/h), and was treated with a calcium channel blocker (amlodipine 10 mg) and an angiotensin II receptor blocker (olmesartan 20 mg). He was observed, but the pain intensified and the hemorrhage spread from the lower pole of the right kidney to the inguinal region, so it was decided that conservative management was not sufficient, and he was transferred to our hospital.

On admission, the patient was 179 cm tall and weighed 86 kg. His blood pressure was 132/79 mmHg and heart rate, 112 bpm. The results of laboratory tests were as follows: hemoglobin was 13,9 g/dL; albumin, 3.8 mg/dL; blood urea nitrogen, 21 mg/dL; creatinine, 1.3 mg/dL; estimated glomerular filtration rate, 48.8 mL/min/1.73 m^2^; prothrombin time, 89% (reference range, 70%-130%), activated partial thromboplastin time, 32.1 s (reference range, 24–36 s); and urinary protein excretion, 0.2 g/day. The urinary erythrocyte sediment contained less than 1 per high-power field. Cerebral aneurysm was ruled out by magnetic resonance angiography.

At the time of the renal hemorrhage, the Hb level was 15.5 g/dL.One week later, upon transfer to our hospital, the Hb level had decreased to 13.8 g/dL prior to TAE. As previously mentioned, renal hemorrhage beneath the renal capsule can compress the renal parenchyma from the outside, leading to a condition known as Page kidney, which is characterized by high-renin hypertension and requires urgent treatment such as surgical nephrectomy or TAE [1–5]. Based on this, we opted for TAE over nephrectomy.

**Fig. 1:**
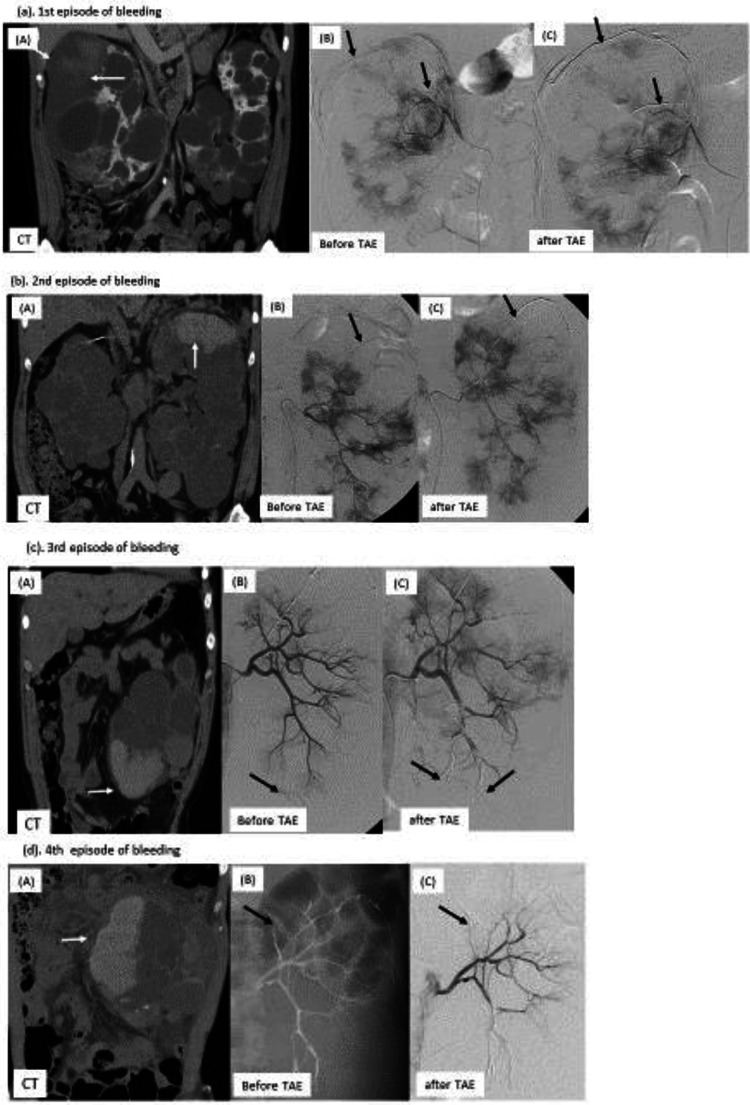
**a** First bleeding episode A: Coronal computed tomography (CT) showed bleeding into the right subcapsular space (arrow). B: Right renal angiogram showed bleeding from the right capsular artery branch (arrow). C: Renal transarterial embolization (TAE) with four microcoils (arrow) was performed on the right capsular artery branch. **b**: Second bleeding episode A: CT showed bleeding into the subcapsular space of the upper pole of the left kidney. B: Left renal angiogram showed bleeding from the left superior capsular artery branch (arrow). C: Renal TAE with four microcoils (arrow) was performed on the left superior capsular artery branch (arrow).**c**: Third bleeding episode A: CT showed bleeding into the subcapsular space of the inferior pole of the left kidney (arrow). B: Left renal angiogram showed bleeding from the inferior pole branch of the left renal artery (arrow). C: Renal TAE with four microcoils (arrow) was performed on the inferior pole branch of the left renal artery. **d**: Fourth bleeding episode A: CT showed bleeding into the subcapsular space in the medial part of the upper pole of the left kidney (arrow). B: Left renal angiogram showed bleeding from the medial part branch of the upper pole of the left kidney. C: Renal TAE with four microcoils (arrow) was performed on the medial part branch of the upper pole of the left kidney

After admission, bleeding into the right subcapsular space continued, so a right renal angiogram was immediately performed according to a previously reported method [6, 7]. Bleeding from the right capsular artery branch was confirmed, and renal TAE was immediately performed with four microcoils, after which hemostasis was confirmed (Fig. 1a). TAE stopped the progression of anemia and improved the pain, and subsequently, renal function decline no longer progressed. On admission day 20, the patient was discharged. Renin activity at discharge normalized to 0.3 (0.2–2.30 ng/mL/hr).

At age 50, the patient developed left-sided flank pain, and bleeding was identified in the subcapsular space of the upper pole of the left kidney. Since the condition worsened during follow-up observation in the first episode, TAE was immediately selected this time. Renal TAE was immediately performed with 4 microcoils on the left superior capsular artery branch, and hemostasis was achieved (Fig. 1b).

Four months later, bleeding was observed in the subcapsular space of the inferior pole of the left kidney. The bleeding was diagnosed as coming from the inferior pole branch of the left renal artery and was stopped with 5 microcoils (Fig. 1c).

At the age of 52 years, bleeding developed into the subcapsular space in the medial part of the upper pole of the left kidney. Renal TAE was performed with 2 microcoils, and hemostasis was achieved (Fig. 1d).

Five months after the fourth renal TAE, renal function progressively declined and hemodialysis was started. One year later, renal hemorrhage developed in both kidneys. Renal TAE was performed to occlude all bilateral renal arteries.

At the time of writing this manuscript, 4 years after the fifth renal TAE, no further bleeding has occurred. During the entire clinical course, no bleeding complications were seen outside the kidneys.

Genetic testing for Ehlers-Danlos syndrome was performed because of the patient’s tendency to bleed, but no genetic abnormality was identified.

## Renal function associated with each renal hemorrhage and subsequent renal TAE

**Fig. 2:**
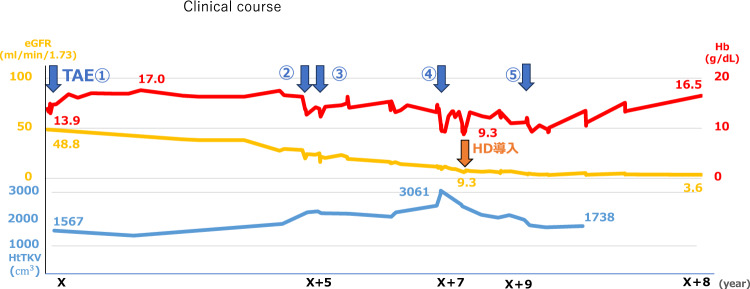
Clinical course of renal function, hemoglobin levels, and total kidney volume (TKV)

Four renal hemorrhages and subsequent changes in renal function related to renal TAE, etc. were investigated (Table 1). Mayo classification at age 45 was equivalent to class 1D.There was no progression of renal function decline before or after the 4 renal bleeding events. The question arises whether treatment with contrast media in such patients with impaired renal function may induce renal dysfunction. Although we performed contrast-enhanced renal TAE, we confirmed that it had no effect on the remaining renal area and that embolization was necessary only at the original bleeding site. Over the course of 8 years, renal size increased over time and renal function was shown to worsen over time (Table 1) (Fig. 2).

**Table 1:** Four renal hemorrhages and each factor before and after

Episode of bleeding		1st	2nd	3rd	4th
Age(years)	40	45	50	50	52
Site of bleeding	Unknown	Upper lateral right kidney	Upper left kidney	Lower left kidnry	Upper medial left kidney
Serum Cre(mg/dL)(before TAE)	1	1.3	2.89	2.79	5.8
Serum Cre(mg/dL)(after TAE)		1.1	2.4	2.65	4.55
eGFR(mL/min/1.73m2)(before TAE)	67.3	48.8	19.8	20.5	9.1
eGFR(mL/min/1.73m2)(after TAE)		58.2	24.2	21.7	11.8
Total kidney volume(cm3)	1260	1567	2254	2218	3061
Hb(before TAE) (g/dL)		13.9	12.7	12.3	9.5
Hb(after TAE) (g/dL)		15.5	16.3	13	10.6
Urinary erythrocyte sediment(/HPF)(before TAE)		< 1	< 1	1–4	< 1

‘Before TAE’ is the day before TAE, and ‘after TAE’ is one week after TAE

The four TKV measurements were all performed at our hospital immediately before TAE, including the bleeding site. The total kidney volume was 1260 cm^3^ on a CT scan performed five years prior to this hospitalization.The eGFR slope/year and ΔTKV/year before the first TAE was 3.7 and 313 cm3. eGFR slope between 1st TAE and 4th TAE was5.6, and ΔTKV/year was 213cm3. Although there was no progression of renal dysfunction immediately after TAE, renal enlargement and renal dysfunction progressed steadily through the four episodes of renal hemorrhage.Since the onset of the first renal hemorrhage, the eGFR slope has tended to decline at a faster rate than before the hemorrhage. However, the progression of ΔTKV/year is slow.

Tolvaptan was started after the first episode of renal hemorrhage, but it was discontinued within a month due to gastric discomfort, so the efficacy of tolvaptan could not be evaluated.

## Discussion

We presented the case of a 45-year-old man with ADPKD who developed spontaneous retroperitoneal hemorrhages with hypertension(equivalent to a Page Kidney) without trauma or other triggers. Subcapsular and perirenal hematomas usually occur after kidney biopsy or trauma, and spontaneous cases are rarely reported. Agarwal et al. reported a case of bilateral subcapsular and perinephric hemorrhage in which renal arteriography revealed multiple aneurysm formation; the patient was eventually diagnosed with polyarteritis nodosa [8]. The group performed renal artery embolization to stop the bleeding but were unable to save the patient because the bleeding spread to other organs. In their article, the authors discuss the importance of early medical treatment. Subcapsular and perinephric hemorrhage has also been reported as spontaneous retroperitoneal hemorrhage. The most common causes are renal cell carcinoma and angiomyolipoma, polyarteritis nodosa, and nephritis. Spontaneous retroperitoneal hemorrhage is characterized by acute onset of nontraumatic subcapsular and perirenal hematomas and is classically characterized by Lenk’s triad: acute flank pain, abdominal tenderness, and signs of internal bleeding [9].

Patients with ADPKD are reported to have a high incidence of renal hemorrhage, most often into a cyst or the renal urinary tract. To our knowledge, only two reports have described subcapsular and perinephric hemorrhage, but in both of them, the bleeding was usually unilateral [10, 11]. In both reports, the authors recommended arterial embolization as a treatment.

We investigated the causes of renal hemorrhage in this case. There were no thrombocytopenia or coagulation abnormalities. Blood pressure had been managed within the normal range with antihypertensive agents for the five years prior to admission, so inadequate blood pressure control was not the cause. There were no episodes of trauma that could have triggered the hemorrhage.We considered the possibility of a genetic disorder such as Ehlers-Danlos syndrome but ruled it out. In ADPKD, renal hemorrhage has been reported as a complication of angiomyolipoma [12], but its presence was not evident on CT. Among cases of TAE performed in ADPKD, some included cases where hemorrhage was the indication for TAE, but only a few cases identified the cause of hemorrhage [13].

Renal hemorrhage is a common complication in ADPKD, but in most cases, bleeding stops spontaneously with conservative management. Surgical nephrectomy or renal TAE is performed in the following two conditions: when bleeding into the renal urinary tract causes bladder tamponade and a renal arteriovenous fistula is suspected, or when subcapsular hemorrhage is associated with a page kidney, as in the present case.Subcapsular renal hemorrhage, as described earlier, causes high-renin hypertension by compressing the renal parenchyma from the outside. This condition is referred to as a “page kidney” and has been reported to require urgent treatment (such as surgical nephrectomy or TAE) [14]. Based on this, we opted for TAE over nephrectomy.

We performed TAE as a temporary treatment for recurrent subcapsular hemorrhage, but this treatment did not completely preserve renal function in this case, and the patient required dialysis approximately eight years later.We performed TAE immediately after hemorrhage because we anticipated that the hemorrhage would spread and affect a larger area of the kidney.Given that the age at dialysis initiation for ADPKD patients in Japan is 63–65 years [15], the initiation of dialysis at age 52 in this case represents early dialysis initiation.Renal hemorrhage may have served as a factor contributing to the progression of renal failure, as evidenced by reports from Suwabe et al., who demonstrated in multiple cases that renal hemorrhage not only causes renal enlargement in ADPKD patients but also acts as a factor contributing to the progression of renal failure [16].

In summary, we experienced a case of bilateral subcapsular and perirenal hematoma that could be treated without dialysis for about 8 years by selective use only of renal TAE in the bleeding branch. In this case, four major bleeds did not cause renal dysfunction, but repeated minor bleeds that did not require renal TAE increased the overall size of the kidney, indicating that renal function may be compromised.
